# Reverse shock index multiplied by simplified motor score as a predictor of clinical outcomes for patients with COVID-19

**DOI:** 10.1186/s12873-024-00948-5

**Published:** 2024-02-14

**Authors:** Meng-Yu Wu, Yueh-Tseng Hou, Jui-Yuan Chung, Giou-Teng Yiang

**Affiliations:** 1 Department of Emergency Medicine, Taipei Tzu Chi Hospital, Buddhist Tzu Chi Medical Foundation, New Taipei, 231 Taiwan; 2 Department of Emergency Medicine, School of Medicine, Tzu Chi University, Hualien, 970 Taiwan; 3 Graduate Institute of Injury Prevention and Control, Taipei Medical University, Taipei, Taiwan; 4 Department of Emergency Medicine, Cathay General Hospital, Taipei, Taiwan; 5 School of Medicine, Fu Jen Catholic University, Taipei, Taiwan; 6 School of Medicine, National Tsing Hua University, Hsinchu, Taiwan

**Keywords:** COVID-19, Shock index, Reverse shock index combined with the Glasgow Coma Scale, Reverse shock index combined with the GCS motor subscale, Reverse shock index combined with the simplified motor score

## Abstract

**Background:**

The reverse shock index (rSI) combined with the Simplified Motor Score (sMS), that is, the rSI-sMS, is a novel and efficient prehospital triage scoring system for patients with COVID-19. In this study, we evaluated the predictive accuracy of the rSI-sMS for general ward and intensive care unit (ICU) admission among patients with COVID-19 and compared it with that of other measures, including the shock index (SI), modified SI (mSI), rSI combined with the Glasgow Coma Scale (rSI-GCS), and rSI combined with the GCS motor subscale (rSI-GCSM).

**Methods:**

All patients who visited the emergency department of Taipei Tzu Chi Hospital between January 2021 and June 2022 were included in this retrospective cohort. A diagnosis of COVID-19 was confirmed through a SARS-CoV-2 reverse-transcription polymerase chain reaction test or SARS-CoV-2 rapid test with oropharyngeal or nasopharyngeal swabs and was double confirmed by checking *International Classification of Diseases, Tenth Revision, Clinical Modification* codes in electronic medical records. In-hospital mortality was regarded as the primary outcome, and sepsis, general ward or ICU admission, endotracheal intubation, and total hospital length of stay (LOS) were regarded as secondary outcomes. Multivariate logistic regression was used to determine the relationship between the scoring systems and the three major outcomes of patients with COVID-19, including. The discriminant ability of the predictive scoring systems was investigated using the area under the receiver operating characteristic curve, and the most favorable cutoff value of the rSI-sMS for each major outcome was determined using Youden’s index.

**Results:**

After 74,183 patients younger than 20 years (*n* = 11,572) and without COVID-19 (*n* = 62,611) were excluded, 9,282 patients with COVID-19 (median age: 45 years, interquartile range: 33–60 years, 46.1% men) were identified as eligible for inclusion in the study. The rate of in-hospital mortality was determined to be 0.75%. The rSI-sMS scores were significantly lower in the patient groups with sepsis, hyperlactatemia, admission to a general ward, admission to the ICU, total length of stay ≥ 14 days, and mortality. Compared with the SI, mSI, and rSI-GCSM, the rSI-sMS exhibited a significantly higher accuracy for predicting general ward admission, ICU admission, and mortality but a similar accuracy to that of the rSI-GCS. The optimal cutoff values of the rSI-sMS for predicting general ward admission, ICU admission, and mortality were calculated to be 3.17, 3.45, and 3.15, respectively, with a predictive accuracy of 86.83%, 81.94%%, and 90.96%, respectively.

**Conclusions:**

Compared with the SI, mSI, and rSI-GCSM, the rSI-sMS has a higher predictive accuracy for general ward admission, ICU admission, and mortality among patients with COVID-19.

**Supplementary Information:**

The online version contains supplementary material available at 10.1186/s12873-024-00948-5.

## Introduction

In March 2020, COVID-19 posed a major threat to health-care systems worldwide. Many emergency departments (EDs) faced challenges in managing patients with COVID-19 while attempting to early identify high-risk populations. The rapid spread of the disease caught hospitals by surprise, and the limited availability of laboratory screening tests, even those with low sensitivity, high costs, and delayed results, rendered identification of high-risk patients challenging. A rapid deposition strategy was required to efficiently manage the large influx of patients.

The shock index (SI) is a physiological scoring system used for triaging patients that is calculated by dividing heart rate (HR) by systolic blood pressure (SBP). This index reflects the hemodynamic status of patients with shock, which is associated with high mortality rates [1]. It can also reliably predict mortality among patients with septic shock. According to a meta-analysis of 8 studies involving 4,557 patients, a high SI score is associated with an increased risk of intensive care unit (ICU) admission, in-hospital mortality, 30-day mortality, and overall mortality [2].

Despite its benefits, the SI has certain limitations in some patient populations and may underestimate the severity of shock because it relies on SBP alone for scoring. Liu et al. [3] used a modified SI (mSI), which incorporates mean arterial pressure (MAP) instead of SBP, and reported that it provides a more accurate assessment of a patient’s hemodynamic status. In a retrospective cohort study, Kurt et al. [4] reported that the mSI scores for in-hospital mortality and ICU admission exhibited an area under the receiver operating characteristic curve (AUROC) of 0.739 and 0.729, respectively.

The rSI-GCS is a newly developed triage scoring system that combines the reverse SI (rSI) with the Glasgow Coma Scale (GCS) to provide a comprehensive assessment of patients’ hemodynamic status and neurological conditions. This system has increased accuracy for predicting shock because it accounts for hemodynamic status and neurological conditions [5, 6]. It also outperforms other conventional scoring systems in terms of predicting short-term mortality, the need for massive transfusion, and the need for early intervention for patients with trauma [5, 7–10]. In addition, the rSI-GCS can be used to evaluate patients with sepsis [11]. A retrospective study conducted in a Japanese tertiary care hospital indicated that compared with the Modified Early Warning Score and Quick Sequential Organ Failure Assessment (SOFA), the rSI-GCS exhibited higher predictive performance for vasopressor use and mechanical ventilation, with AUROC values of 0.85 and 0.82, respectively [11]. Although the rSI-GCS has strong discriminant ability as an early-phase predictor for patients with suspected sepsis, its application in prehospital settings remains limited because it is complex and time-consuming to implement.

Several tools for evaluating consciousness, such as the three-point simplified motor score (sMS) and the GCS motor subscale (GCSM), have been developed as field triage instruments for promptly identifying patients at a high risk of unfavorable outcomes to ensure such patients are quickly transported to a facility that provides definitive care. Both the sMS and the GCSM have undergone validation and demonstrated accuracy and reliability close to those of the GCS with respect to predicting poor outcomes among patients with intracranial hemorrhage [12–14]. Studies have indicated that the sMS has similar performance to that of the GCS in terms of predicting mortality and the need for endotracheal intubation in EDs [15, 16].

In this study, we enhanced the rSI-GCS by combining the sMS and GCSM to establish the rSI-sMS and rSI-GCSM scales. These scales are easy to use, and their dichotomized cutoff points are useful in field triage procedures because they simplify the criteria for feasibility and emergency medical service training. However, their discriminant ability among patients with COVID-19 requires validation. Therefore, we investigated the predictive accuracy of the rSI-sMS and rSI-GCSM as novel scoring systems for assessing patients with COVID-19 and compared the rSI-sMS with the SI, mSI, and rSI-GCS. We also hypothesized that the rSI-sMS would exhibit high predictive performance for field triage in patients with COVID-19.

## Methods

### Study design and participant selection

This retrospective cohort study was conducted at Taipei Tzu Chi Hospital and was approved by the hospital’s institutional review board (approval number: 10-XD-074, 10-XD-080, 10-XD-151). All patients who visited the ED of Taipei Tzu Chi Hospital between January 2021 and June 2022 were included in the study. In May 2022, Taiwan experienced a rapid influx of patients with COVID-19. On May 25, 2022, an announcement was made by the Central Epidemic Command Center to revise the definition of a confirmed COVID-19 case. This revision, which went into effect on May 26, 2022, indicated that a confirmed case was considered to be one in which an individual of any age or group received a positive COVID-19 test result through an at-home rapid antigen test kit that was subsequently verified by a medical professional. This definition also encompassed individuals with a positive rapid antigen test conducted by a medical professional. In this study, a diagnosis of COVID-19 was confirmed through a SARS-CoV-2 reverse-transcription polymerase chain reaction test or SARS-CoV-2 rapid test through oropharyngeal or nasopharyngeal swabs and double-confirmed *International Classification of Diseases, Tenth Revision, Clinical Modification* (*ICD-10-CM*) codes in electronic medical records. Patients younger than 20 years were excluded because their vital signs differ from those of the adult population.

### Variable measurements

#### Field triage measures

First proposed by Allgöwer and Burri in 1967 [17], the SI is calculated by dividing HR by SBP. Patients in shock often experience an increase in HR and a decrease in SBP. The normal SI range for a healthy adult typically ranges from 0.5 to 0.7, with an SI of ≥ 1.0 indicating shock status [18]. The SI is widely used in emergency care for patients with sepsis; patients with other critical conditions, such as trauma [19, 20], acute coronary syndrome [21, 22], and postpartum hemorrhage [23, 24]; and older patients [25, 26].

In the mSI, MAP, which is calculated using diastolic blood pressure (DBP), is used instead of SBP, and therefore, the mSI has stronger predictive ability (mSI = HR/MAP). A prospective observational study conducted at a tertiary care teaching hospital indicated that the mSI exhibited adequate predictive validity in predicting the need for mechanical ventilation after 24 h [27]. Similar findings were reported by Althunayyan et al., [28] who indicated that the mSI is an effective predictor of sepsis, ICU admission, and 28-day mortality when a cutoff value of over 1 is used.

The rSI-GCS is an index that combines the SI and GCS and is calculated as follows: rSI-GCS = 1/SI × GCS [5]. The GCS is commonly used to evaluate a patient’s level of consciousness and is a stronger predictor of mortality than SBP, respiratory rate (RR), and age [29, 30]. The rSI-GCS is a useful and accurate triage tool for detecting hypovolemic shock and can also be used to predict the need for massive blood transfusion, coagulopathy, in-hospital mortality, and 24-h mortality in patients with trauma [7, 8, 10, 31–36]. However, utilizing the GCS during prehospital triage is a time-consuming process. The sMS and GCSM exhibit similar predictive performance to that of the total GCS score [12–16]. Therefore, in this study, we modified the rSI-GCS to create the rSI-GCSM and rSI-sMS by using the following formulas: rSI-GCSM = 1/SI × GCSM and rSI-sMS = 1/SI × sMS. We also investigated the accuracy and predictive performance of various scoring systems, including the SI, mSI, rSI-GCS, rSI-GCSM, and rSI-sMS, for patients with COVID-19.

#### Covariates

We examined the basic characteristics of patients with COVID-19, including their age, sex, underlying diseases, vital signs, GCS scores, sMS scores, and sepsis severity. We also recorded their vital signs, including their HR, SBP, DBP, and RR, once they arrived at the hospital, and we used these values to calculate prediction scores. Systemic inflammatory response syndrome (SIRS) criteria and SOFA scores were used to diagnose sepsis. Hyperlactatemia was defined as a lactate concentration exceeding 2 mmol/L, and severe hyperlactatemia was defined as a lactate concentration exceeding 4 mmol/L. In critically ill patients or those experiencing shock or hypoperfusion, the levels of lactate often exceed 2 mmol/L. Patients with lactate levels exceeding 4 mmol/L typically require immediate resuscitation and ICU admission [1–5]. In this study, we selected a cutoff value of 4 mmol/L as a predictive factor of severe hyperlactatemia [37–41]. We also categorized patients aged 65 or older as older patients in our subgroup analysis.

### Outcomes

In-hospital mortality was regarded as the primary outcome of this study, and sepsis, general ward or ICU admission, endotracheal intubation, and total hospital length of stay (LOS) were regarded as secondary outcomes. A diagnosis of sepsis was established using SOFA scores in accordance with the Third International Consensus Definitions for Sepsis and Septic Shock guidelines [42]. Sepsis diagnoses were confirmed by cross-referencing *ICD-10-CM* codes and electronic medical records obtained after ED discharge.

### Statistical analysis

Demographic data, injury-related data, and clinical outcomes were analyzed using IBM SPSS Statistics, version 20.0 (IBM, Armonk, NY, USA). Continuous data with a normal distribution were analyzed using the Kolmogorov–Smirnov test. Continuous variables with a normal distribution are presented as means (standard deviations), and nonnormal variables are presented as medians (interquartile ranges). Categorical variables are presented as numbers and percentages. Continuous variables with a nonnormal distribution were nonparametrically examined using analysis of variance or Mann–Whitney *U* tests, and categorical and nominal variables were analyzed using Pearson’s chi-squared test or Fisher’s exact test. Multivariate logistic regression was used to determine the relationship between the scoring systems and the clinical outcomes of patients with COVID-19. Variables with *p* values less than 0.10 in the chi-squared test or Mann–Whitney *U* test were included in the multivariate logistic regression analysis by using the forced entry method.

The AUROC was calculated for each outcome to evaluate the discriminative ability of the five predictive scoring systems. The differences between the AUROC values were assessed by comparing the AUROC values of any two scoring systems. Delong’s test was conducted to compare the AUROC values of all scoring systems. Youden’s index (sensitivity + specificity − 1) was used to determine the most favorable cutoff value for the rSI-sMS for each major outcome. Sensitivity, specificity, positive predictive value, negative predictive value, the positive likelihood ratio, the negative likelihood ratio, and accuracy were calculated on the basis of the optimal cutoff value. Patients who had missing vital sign records were excluded from the analysis. All tests were two-sided, with statistical significance set at *p* < 0.05.

## Results

### Participant characteristics

**Fig. 1:**
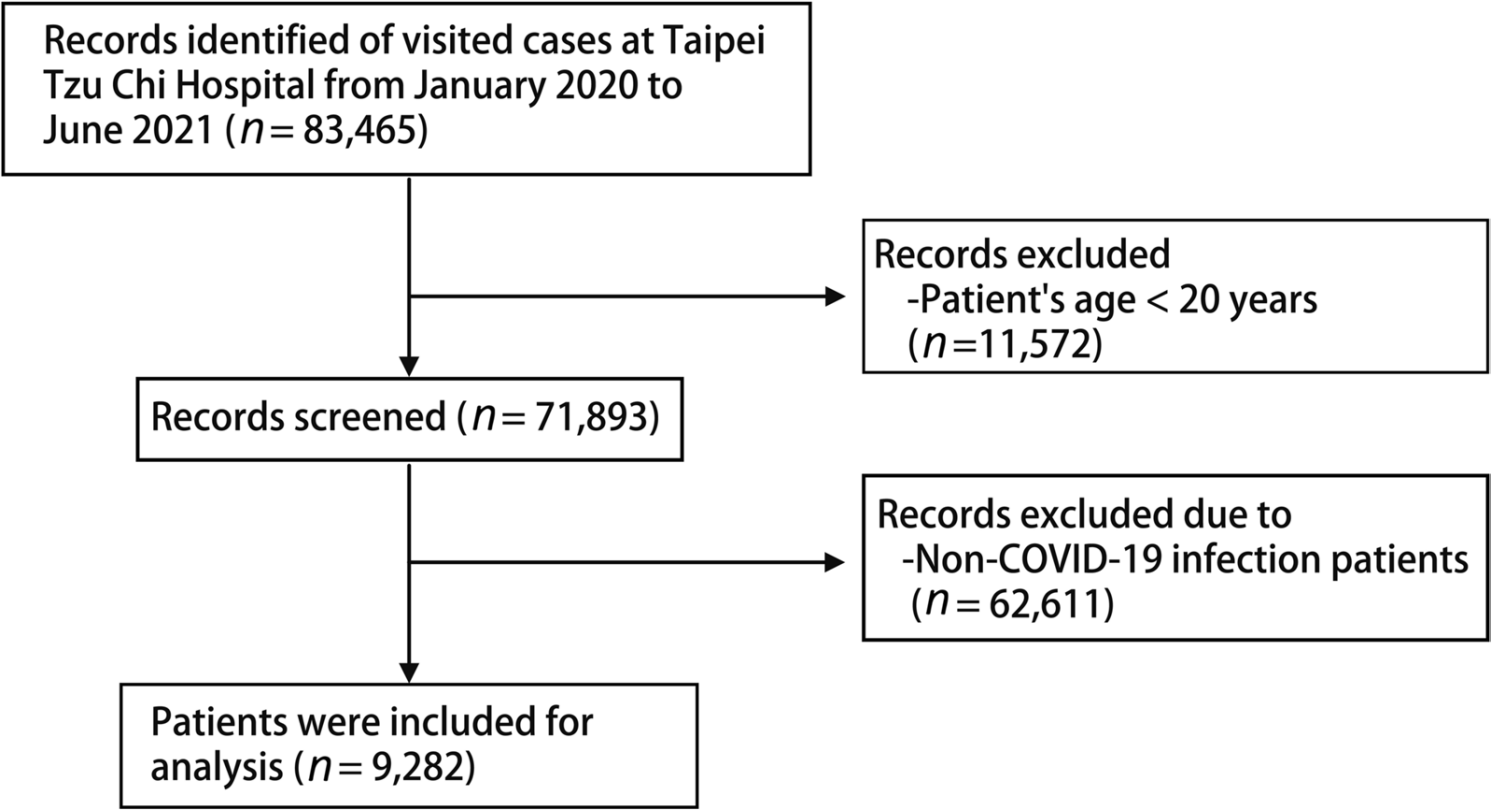
Flowchart of patient inclusion

After 74,183 patients aged younger than 20 years (*n* = 11,572) and without COVID-19 (*n* = 62,611) were excluded, 9,282 patients with COVID-19 (median age: 45 years, interquartile range: 33–60 years, 46.1% men) were identified as eligible for inclusion in the study (Fig. 1). The rate of in-hospital mortality was determined to be 0.75% (Table 1). The main underlying disease was identified as a central nervous system disease. Compared with the groups of patients admitted to general wards and ICUs, the mortality group comprised a larger proportion of individuals aged older than 65 years (85.7%), with the median age of the group being 80 years. The mortality group also comprised a larger proportion of individuals with SIRS scores of ≥ 2, SOFA scores of ≥ 2, and severe hyperlactatemia. By contrast, the proportions of individuals with SIRS scores of ≥ 2 were similar between the groups of patients who were admitted to general wards and those who were admitted to ICUs. In terms of clinical outcomes, the mortality group exhibited a higher rate of admission to general wards and ICUs but a shorter total LOS.

**Table 1:** Demographic characteristics of patients

**Characteristics**	Total	Admission	ICU admission	Mortality
Patient number	9282(100%)	660(7.11%)	85(0.92%)	70(0.75%)
Age (years)				
Age [median(IQR)]	45(33–60)	68(52–81)	69(55.5–80.5)	80(70–88)
Age < 65ys	7643(82.3%)	289(43.8%)	36(42.4%)	10(14.3%)
Age ≥ 65ys	1639(17.7%)	371(56.2%)	49(57.6%)	60(85.7%)
Sex, n (%)				
Female	5002(53.9%)	319(48.3%)	31(36.5%)	27(38.6%)
Male	4280(46.1%)	341(51.7%)	54(63.5%)	43(61.4%)
Vital sign				
SBP	139.32 ± 32.95	138.32 ± 26.45	133.68 ± 27.67	137.50 ± 27.33
DBP	79.79 ± 108.16	75.14 ± 14.68	73.01 ± 16.02	75.70 ± 18.01
HR	96.64 ± 18.16	98.06 ± 20.66	102.30 ± 22.32	108.15 ± 22.07
RR	18.66 ± 8.12	19.78 ± 5.59	21.45 ± 4.56	23.20 ± 4.94
Temperature	37.06 ± 3.91	33.84 ± 1.77	33.45 ± 1.11	35.79 ± 0.76
Saturation	97.25 ± 18.74	94.89 ± 5.43	89.77 ± 11.19	89.24 ± 10.74
GCS score	15(15–15)	15(15–15)	15(10–15)	11(7–15)
Score systems				
Shock index (SI)	0.91 ± 0.30	0.89 ± 0.32	0.91 ± 0.29	0.88 ± 0.32
mSI	1.02 ± 0.18	1.05 ± 0.27	1.11 ± 0.38	1.17 ± 0.38
rSI-GCS	22.28 ± 7.37	20.81 ± 7.56	18.36 ± 9.70	15.02 ± 7.35
rSI-GCS M	8.92 ± 2.94	8.42 ± 3.00	7.58 ± 3.88	6.25 ± 2.98
rSI-sMS	4.44 ± 1.50	4.10 ± 1.62	3.64 ± 2.03	2.82 ± 1.64
Sepsis				
SIRS score ≥ 2	5812(62.6%)	407(61.7%)	57(67.1%)	51(72.9%)
SOFA score ≥ 2	160(1.7%)	122(18.5%)	28(32.9%)	27(38.6%)
Lactate ≥ 4.0	22(0.2%)	22(3.3%)	7(8. 2%)	16(22.9%)
Comorbidity				
CNS diseases	230(2.5%)	112(17.0%)	16(18.8%)	16(22.9%)
CVD	55(0.6%)	11(1.7%)	2(2.4%)	1(1.4%)
CKD	68(0.7%)	33(5.0%)	7(8.2%)	6(8.6%)
Diabetes mellitus	117(1.3%)	53(8.0%)	8(9.4%)	6(8.6%)
Outcomes				
Admission	660(7.11%)	––	77(90.6%)	61(87.1%)
ICU admission	85(0.92%)	77(11.7%)	––	26(37.1%)
ED death	9(0.1%)	––	––	9(12.9%)
In-hospital death	70(0.75%)	61(9.2%)	26(30.6%)	––
Total LOS days	9(5–15)	9(5–15)	17(10–26)	6(3–14.5)

*HR* heart rate, *SBP* systolic blood pressure, *DBP* diastolic blood pressure, *RR* respiratory rate, *GCS* Glasgow Coma Scale, *SI* shock index, *mSI* modified shock index, *rSI-GCS* reverse shock index combined with the Glasgow Coma Scale, *rSI-GCSM* reverse shock index combined with the Glasgow Coma Scale motor subscale, *rSI-sMS* reverse shock index combined with the simplified motor score, *CNS* central nervous system, *CKD* chronic kidney disease, *CVD* cardiovascular disease, *SIRS* systemic inflammatory response syndrome, *SOFA* Sequential Organ Failure Assessment, *ED* emergency department, *LOS* length of stay, *ICU* intensive care unit

### Subgroup analysis of SI, mSI, rSI-GCS, rSI-GCSM, and rSI-sMS

The SI and mSI scores were significantly high in patients with sepsis (SIRS scores of ≥ 2 or SOFA scores of ≥ 2), patients admitted to general wards, patients admitted to ICUs, and the mortality group (Table 2), but they did not differ between patients with severe hyperlactatemia and patients with a total LOS of ≥ 14 days. By contrast, the rSI-GCS, rSI-GCSM, and rSI-sMS scores were significantly low in patients with sepsis, patients with hyperlactatemia, patients admitted to general wards, patients admitted to ICUs, patients with a total LOS of ≥ 14 days, and the mortality group.

**Table 2:** Subgroup analysis of the SI, mSI, rSI-GCS, rSI-GCSM, and rSI-sMS

Subgroups	SI		mSI		rSI-GCS		rSI-GCSM		rSI-sMS	
	Mean ± SD	*P*-value	Mean ± SD	*P*-value	Mean ± SD	*P*-value	Mean ± SD	*P*-value	Mean ± SD	*P*-value
Sepsis										
SIRS score < 2	0.59 ± 0.12	< 0.001	0.84 ± 0.16	< 0.001	26.55 ± 8.03	< 0.001	10.62 ± 3.18	< 0.001	5.30 ± 1.59	< 0.001
SIRS score ≥ 2	0.78 ± 0.15		1.10 ± 0.21		19.79 ± 5.59		7.92 ± 2.23		3.95 ± 1.13	
SOFA score < 2	0.71 ± 0.17	< 0.001	1.00 ± 0.23	< 0.001	22.37 ± 7.32	< 0.001	8.96 ± 2.91	< 0.001	4.47 ± 1.45	< 0.001
SOFA score ≥ 2	0.81 ± 0.25		1.17 ± 0.33		17.43 ± 8.27		7.11 ± 3.32		3.35 ± 1.79	
Lactatemia										
Lactate < 4.0	0.81 ± 0.25	0.131	1.16 ± 0.32	0.140	17.50 ± 7.60	0.005	7.19 ± 3.01	0.002	3.36 ± 1.67	0.002
Lactate ≥ 4.0	0.88 ± 0.19		1.25 ± 0.25		13.41 ± 5.92		5.38 ± 2.37		2.43 ± 1.19	
General ward admission										
Non-admission	0.71 ± 0.17	< 0.001	1.00 ± 0.23	< 0.001	22.40 ± 7.34	< 0.001	8.96 ± 2.92	< 0.001	4.48 ± 1.46	< 0.001
Admission	0.73 ± 0.22		1.05 ± 0.28		20.83 ± 7.55		8.43 ± 2.97		4.10 ± ± 1.60	
ICU										
Non-admission	0.71 ± 0.17	0.003	1.00 ± 0.23	< 0.001	22.32 ± 7.33	0.001	8.93 ± 2.92	0.003	4.45 ± 1.46	0.001
Admission	0.80 ± 0.24		1.13 ± 0.30		18.42 ± 9.66		7.57 ± 3.86		3.65 ± 2.00	
Total LOS day										
Days < 14	0.73 ± 0.22	0.083	1.04 ± 0.30	0.410	21.40 ± 7.98	0.002	8.66 ± 3.14	0.001	4.22 ± 1.68	0.002
Days ≥ 14	0.76 ± 0.19		1.06 ± 0.25		19.55 ± 6.35		7.91 ± 2.48		3.83 ± 1.37	
Mortality										
Survival	0.71 ± 0.16	0.001	1.00 ± 0.23	< 0.001	22.34 ± 7.34	< 0.001	8.94 ± 2.92	< 0.001	4.46 ± 1.46	< 0.001
Death	0.82 ± 0.23		1.16 ± 0.32		14.97 ± 7.32		6.29 ± 2.94		2.82 ± 1.59	

*SD* standard deviation, *SI* shock index, *mSI* modified shock index, *rSI-GCS* reverse shock index combined with the Glasgow Coma Scale, *rSI-GCSM* reverse shock index combined with the Glasgow Coma Scale motor subscale, *rSI-sMS* reverse shock index combined with the simplified motor score, *SIRS* systemic inflammatory response syndrome, *SOFA* Sequential Organ Failure Assessment, *LOS* length of stay, *ICU* intensive care unit

### Predictive accuracy for clinical outcomes

**Fig. 2:**
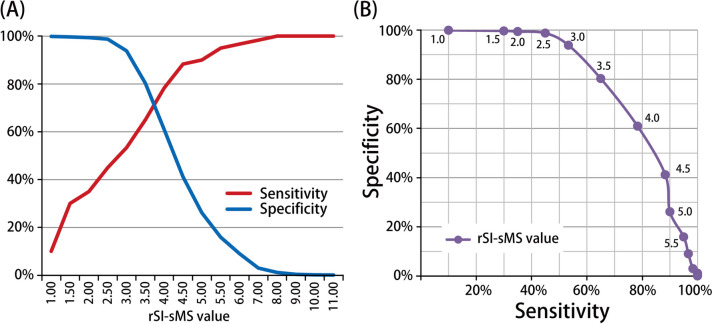
Sensitivity and specificity of the rSI-sMS for each cutoff value for mortality

The rSI-GCS, rSI-GCSM, and rSI-sMS scores served as significant predictors of general ward admission and mortality. The SI and mSI score only served as a significant predictor of general ward admission (Table 3). As indicated in Table 4, the rSI-sMS score exhibited a similar predictive accuracy to that of the rSI-GCS score for all outcomes and a higher predictive accuracy than that of the rSI-GCSM score. The most favorable cutoff values for the rSI-sMS for predicting general ward admission, ICU admission, and mortality among patients with COVID-19 were 3.17, 3.45, and 3.15, respectively. As presented in Table 5, the predictive performance of the rSI-sMS for mortality was associated with a sensitivity of 60% and a specificity of 91%, whereas the predictive performance of the rSI-sMS (≥ 3.17) for hospital admission was associated with a sensitivity of 25% and a specificity of 92%. In terms of ICU admission, the predictive performance of the rSI-sMS (≥ 3.45) was associated with a sensitivity of 49% and a specificity of 82%. Figure 2 presents the sensitivity and specificity of the rSI-sMS for each cutoff value for mortality.

**Table 3:** Clinical outcome prediction using multivariate logistic regression

Scoring systems	aOR of death			aOR of admission			aOR of ICU admission		
	aOR	95% CI	*p*–Value	aOR	95% CI	*p*–Value	aOR	95% CI	*p*–Value
SI	1.68	0.35–8.07	0.517	5.37	2.81–10.3	< 0.001	2.00	0.54–7.61	0.291
mSI	1.17	0.35–3.87	0.802	2.98	1.88–4.72	< 0.001	1.51	0.56–4.12	0.417
rSI-GCS	0.90	0.84–0.96	0.002	0.95	0.93–0.97	< 0.001	0.99	0.96–1.03	0.730
rSI-GCSM	0.81	0.69–0.95	0.008	0.88	0.84–0.93	< 0.001	0.99	0.92–1.08	0.918
rSI-sMS	0.67	0.50–0.89	0.006	0.76	0.69–0.84	< 0.001	1.01	0.89–1.15	0.862

The covariables used in the multivariate logistic regression included age, sex, cardiovascular disease, central nervous system disease, chronic kidney disease, diabetes mellitus, systemic inflammatory response syndrome (SIRS) score, Quick Sequential Organ Failure Assessment (qSOFA) score, and Sequential Organ Failure Assessment (SOFA) score

Each scoring system was separately evaluated using multivariable logistic regression because of their strong collinearity

*aOR* adjusted odds ratio, *CI* confidence interval, *SI* shock index, *mSI* modified shock index, *aOR* adjusted odds ratio, *rSI-GCS* reverse shock index combined with the Glasgow Coma Scale, *rSI-GCSM* reverse shock index combined with the Glasgow Coma Scale motor subscale, *rSI-sMS* reverse shock index combined with the simplified motor score

**Table 4:** Comparison of AUROCs with the SI, mSI, rSI-GCS, rSI-GCSM, and rSI-sMS for the prediction of sepsis, endotracheal intubation, general ward admission, ICU admission, and mortality

Scoring systems	AUROC	**Difference between areas (95% Confidence Interval)**				
		**SI**	**mSI**	**rSI-GCS**	**rSI-GCSM**	**rSI-sMS**
Predicting sepsis						
SI	0.612(0.559–0.665)***	––	––	––	––	––
mSI	0.655(0.604–0.706)***	0.043(0.029–0.057)***	––	––	––	––
rSI-GCS	0.709(0.656–0.763)***	0.098(0.066–0.129)***	0.054(0.020–0.089)**	––	––	––
rSI-GCSM	0.699(0.645–0.752)***	0.087(0.058–0.116)***	0.044(0.011–0.076)**	0.011(0.002–0.019)**	––	––
rSI-sMS	0.716(0.661–0.770)***	0.104(0.0699–0.138)***	0.061(0.024–0.098)**	0.007(-0.002–0.015)	0.017(0.007–0.027)**	––
Predicting intubation						
SI	0.780(0.697–0.863)***	––	––	––	––	––
mSI	0.776(0.689–0.862)***	0.004(-0.036–0.044)	––	––	––	––
rSI-GCS	0.848(0.767–0.928)***	0.068(0.009–0.127)**	0.072(0.004–0.141)**	––	––	––
rSI-GCSM	0.837(0.757–0.917)***	0.057(-0.001–0.115)	0.061(-0.007–0.130)	0.011(-0.017–0.039)	––	––
rSI-sMS	0.844(0.763–0.925)***	0.064(-0.002–0.130)	0.068(-0.008–0.145)	0.004(-0.032–0.040)	0.007(-0.010–0.024))	––
Predicting admission						
SI	0.525(0.499–0.550)**	––	––	––	––	––
mSI	0.546(0.521–0.571)***	0.021(0.014–0.029)***	––	––	––	––
rSI-GCS	0.575(0.549–0.602)***	0.051(0.038–0.063)***	0.029(0.015–0.044)***	––	––	––
rSI-GCSM	0.567(0.541–0.593)***	0.042(0.031–0.053)***	0.021(0.008–0.034)**	0.008(0.005–0.012)***	––	––
rSI-sMS	0.580(0.553–0.607)***	0.056(0.042–0.069)***	0.034(0.019–0.050)***	0.005(0.001–0.009)**	0.013(0.009–0.018)****	––
Predicting ICU admission						
SI	0.609(0.537–0.681)**	––	––	––	––	––
mSI	0.635(0.565–0.704)***	0.026(0.003–0.049)***	––	––	––	––
rSI-GCS	0.682(0.611–0.754)***	0.073(0.037–0.109)***	0.047(0.006–0.089)***	––	––	––
rSI-GCSM	0.665(0.592–0.737)***	0.056(0.023–0.088)***	0.030(-0.009–0.068)	0.018(0.007–0.028)***	––	––
rSI-sMS	0.681(0.609–0.754)***	0.072(0.035–0.110)***	0.047(0.004–0.089)***	0.001(-0.012–0.014)	0.017 (0.004–0.030)***	––
Predicting mortality						
SI	0.637(0.556–0.718)***	––	––	––	––	––
mSI	0.663(0.585–0.741)***	0.026(0.005–0.047)**	––	––	––	––
rSI-GCS	0.788(0.718–0.859)***	0.151(0.086–0.217)***	0.125(0.0548–0.196)***	––	––	––
rSI-GCSM	0.771(0.700–0.842)***	0.134(0.075–0.193)***	0.108(0.0435–0.173)***	0.017(-0.003–0.038)	––	––
rSI-sMS	0.804(0.733–0.875)***	0.167(0.095–0.239)***	0.141(0.0658–0.216)***	0.016(-0.004–0.035)	0.033(0.009–0.057)***	––

*SI* shock index, *mSI* modified shock index, *rSI-GCS* reverse shock index combined with the Glasgow Coma Scale, *rSI-GCSM* reverse shock index combined with the Glasgow Coma Scale motor subscale, *rSI-sMS* reverse shock index combined with the simplified motor score

Analyzed using Delong’s test: ^*^p < 0.05; ^**^p < 0.001; ^***^p < 0.0001

**Table 5:** Predictive performance of the rSI-sMS for general ward admission, ICU admission, and mortality

Variables	Admission	ICU admission	Inhospital mortality
Cut-off value (95% Cl)	3.17	3.45	3.15
Sensitivity, %	25.04% (21.70%-28.61%)	48.68% (37.04%-60.43%)	60.00% (46.54%-72.44%)
Specificity, %	91.51% (90.89%-92.10%)	82.23% (81.42%-83.02%)	91.17% (90.56%-91.75%)
Positive Likelihood Ratio	2.95 (2.53–3.44)	2.74 (2.17–3.47)	6.79 (5.47–8.44)
Negative Likelihood Ratio	0.82 (0.78–0.86)	0.62 0.50–0.78)	0.44 (0.32–0.60)
Positive predictive value, %	18.27% (15.74%-21.01%)	2.29% (1.62%-3.14%)	4.38% (3.09%-6.01%)
Negative predictive value, %	94.16% (93.62%-94.66%)	99.47% (99.27%-99.62%)	99.71% (99.56%-99.81%)
Accuracy	86.83% (86.11%-87.53%)	81.94% (81.13%-82.74%)	90.96% (90.35%-91.55%)

### Predictive performance of the rSI-sMS in subgroup analysis

**Fig. 3:**
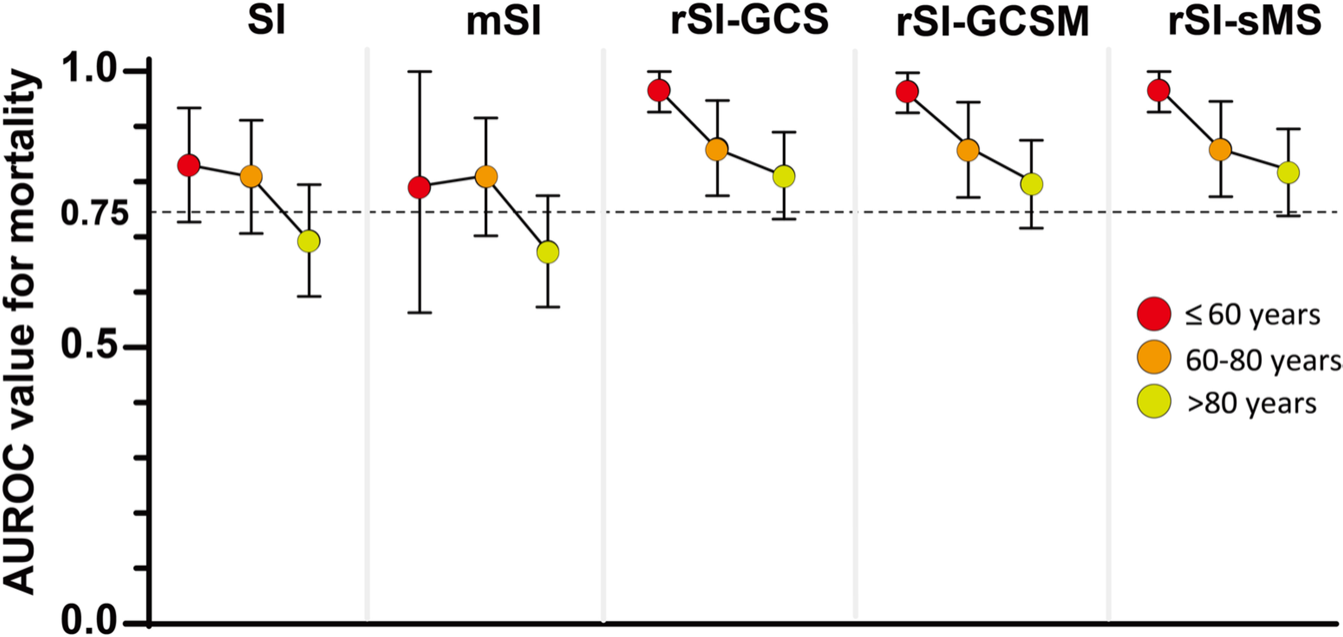
Predictive performance of the five scoring systems with respect to age

As indicated in Table 6, subgroup analysis revealed that the rSI-sMS was associated with a larger AUROC for predicting mortality compared with the SI, mSI, in various subgroups, including those of patients with and without comorbidities, patients with sepsis (SIRS < 2, SIRS ≥ 2, and SOFA < 2), and older and younger patients, with an AUROC of at least 0.77. In all subgroups, the AUROC of the rSI-sMS was similar to that of the rSI-GCS and rSI-GCSM. To evaluate the predictive performance of the five scoring systems with respect to age, three age groups were designated: < 60 years, 60–80 years, and ≥ 80 years. The results indicated that the predictive performance of the SI and mSI for mortality declined with age, with an AUROC of < 0.75 for patients aged younger than 80 years. By contrast, the AUROC values of the rSI-GCS, rSI-GCSM, and rSI-sMS for mortality remained at > 0.75 for all age groups (Fig. 3).

**Table 6:** Subgroup analysis of the predictive performance of the five scoring systems for the prediction of mortality

Scoring systems	Area under the receiver operating characteristic (AUROC)				
	SI	mSI	rSI-GCS	rSI-GCSM	rSI-sMS
Age					
Age < 65 years	0.611(0.357–0.865)	0.621(0.360–0.883)	0.836(0.639–0.999)	0.836(0.639–0.999)	0.835(0.639–0.999)
Age ≥ 65 years	0.748(0.671–0.825)	0.747(0.671–0.822)	0.844(0.783–0.904)	0.832(0.771–0.894)	0.849(0.788–0.910)
Comorbidity					
With comorbidity	0.640(0.506–0.773)	0.641(0.510–0.772)	0.766(0.647–0.884)	0.758(0.638–0.878)	0.779(0.662–0.895)
Without comorbidity	0.681(0.589–0.774)	0.703(0.615–0.791)	0.810(0.731–0.888)	0.792(0.713–0.871)	0.816(0.737–0.895)
Sepsis					
SIRS score < 2	0.442(0.251–0.632)	0.489(0.272–0.705)	0.743(0.548–0.938)	0.728(0.526–0.930)	0.767(0.565–0.968)
SIRS score ≥ 2	0.622(0.534–0.710)	0.648(0.563–0.733)	0.766(0.684–0.849)	0.741(0.657–0.826)	0.777(0.694–0.860)
SOFA score < 2	0.557(0.445–0.670)	0.580(0.475–0.685)	0.761(0.660–0.863)	0.731(0.629–0.833)	0.791(0.689–0.893)
SOFA score ≥ 2	0.628(0.516–0.739)	0.624(0.510–0.738)	0.663(0.545–0.781)	0.661(0.545–0.778)	0.645(0.526–0.764)

*SI* shock index, *mSI* modified shock index, *rSI-GCS* reverse shock index combined with the Glasgow Coma Scale, *rSI-GCSM* reverse shock index combined with the Glasgow Coma Scale motor subscale, *rSI-sMS* reverse shock index combined with the simplified motor score, *CVD* cardiovascular disease, *SIRS* systemic inflammatory response syndrome, *SOFA* Sequential Organ Failure Assessment, *ICU* intensive care unit

## Discussion

Evaluating the risk of mortality in hospital-admitted patients with COVID-19 is key in ensuring intensive observation and timely medical intervention. Developing a rapid and accurate triage tool may help health-care professionals communicate prognoses to the families of their patients and may provide such professionals with a rapid deposition strategy. In this study, we modified and simplified a widely used triage tool, namely the rSI-sMS, the score of which is used to assess COVID-19 mortality risk, to offer a simple and objective method for predicting clinical outcomes in patients with COVID-19, including sepsis, endotracheal intubation, general ward or ICU admission, and mortality. The rSI-sMS accurately predicts sepsis, endotracheal intubation, and mortality, with an AUROC of 0.716 (95% confidence interval [CI]: 0.661–0.770), 0.844 (95% CI: 0.763–0.925), and 0.804 (95% CI: 0.733–0.875), respectively, indicating it has higher predictive performance than the SI and mSI do. Compared with the rSI-GCS and rSI-GCSM, the rSI-sMS is faster, is easier to use, and has higher predictive performance.

Although many modified scoring systems have been developed to improve the accuracy of prediction for patients with COVID-19, few studies have focused on these systems’ predictive accuracy and application in prehospital or ED triage settings. Neto et al. [43] examined 11 risk stratification scores and reported that none of them exhibited a high accuracy (AUROC > 0.80) in predicting in-hospital mortality. They reported that only seven of these scores exhibited an acceptable accuracy (AUROC = 0.75–0.80). Demir et al. [44] examined the performance of various prediction models, including the Pandemic Medical Early Warning Score, Simple Triage Scoring System (STSS), and CURB-65, for patients with COVID-19. They reported that although the predictive performance of these models for 30-day mortality, ICU admission, and mechanical ventilation appeared to be acceptable, the data collected on a routine basis did not typically include information on arterial blood gas [45]. Generally, these models are more complex and more difficult to implement than the rSI-sMS is, and therefore, they are less practical for use in prehospital or triage settings. In prehospital care and ED settings, the rSI-sMS score is easy to calculate; it can be calculated without laboratory or oximetric data. In this study, we provided strong evidence that the rSI-sMS is a useful predictor of mortality in patients with COVID-19. We also reported that the rSI-sMS has a significantly high predictive accuracy for sepsis, endotracheal intubation, general ward admission, and ICU admission among patients with COVID-19.

Although pandemic-control-related restrictions have been lifted and daily life has returned to normal in Taiwan, the government should remain prepared for a potential resurgence of COVID-19 cases among less comorbid populations. Overall, the COVID-19 pandemic has underscored the importance of preserving health-care resources to ensure health-care facilities can continue to provide standard services as they address the requirements of health emergencies such as pandemics. To prevent wastage of resources and funding, accurate triaging of high-risk patients with COVID-19 is essential in resource-limited health-care settings. Although the optimal cutoff values of the rSI-sMS were determined using the highest Youden’s index, obtaining cutoff values with higher sensitivity and acceptable specificity could aid in minimizing undertriage. Therefore, in this study, we determined the sensitivities and specificities associated with various cutoff values for predicting mortality through the rSI-sMS score. At a cutoff value of 4.5, a high sensitivity of 88.3% and an acceptable specificity of 41.7% were obtained. Given that the rSI-sMS score exhibits higher performance in terms of specificity than sensitivity, specificity should be prioritized over sensitivity when the rSI-sMS score is utilized, and therefore, the score may be suitable for resource-limited health-care settings. Furthermore, because oxygen saturation is not incorporated into the rSI-sMS, the tool can be easily implemented in a variety of health-care settings. Patients with COVID-19 with an rSI-sMS score of < 4.5 should receive definitive care in specialized centers.

This study has several strengths. First, to our knowledge, this is the first study to evaluate five major SI-related scoring systems with respect to their ability to predict mortality in patients with COVID-19. Our analysis encompassed several key clinical outcomes. Second, this study included an Asian population; Asian populations have not been extensively explored in previous studies, and the adverse events that frequently occur in Asian and White populations generally differ. Third, this study streamlined the rSI-GCS to establish the rSI-sMS, which is as precise as the rSI-GCS but requires less time to assess.

This study also has some limitations. First, the retrospective nature of this study resulted in some missing clinical data. However, the number of cases with missing values for vital signs and GCS scores was small (321 cases, 3.45%). In addition, imputation was deemed inappropriate for the data obtained from vital sign records. We conducted a comparison of the basic characteristics of the included sample and the sample of individuals who were excluded because of missing data (Supplementary Table 1), and we observed differences in their sepsis status (SIRS score ≥ 2), hyperlactatemia, and clinical outcomes. Notably, the main differences between the two patient samples were related to the outcome variables, including ICU admission, ED mortality, and in-hospital mortality. This discrepancy was presumably due to the excluded patients having presented with severe shock upon arrival; this meant that their vital signs, particularly SBP, could not be measured using machines. Notably, the triage personnel who recorded the vital signs may not have been able to predict the patients’ outcomes, and therefore, a large portion of the missing data can be considered to be missing at random. Although we excluded half of the patient sample from our analysis, our study included more than 5,000 patients. Therefore, the results of this study can be considered valuable and informative and to contribute to addressing the knowledge gap in this area. Nevertheless, we acknowledge that our analysis is susceptible to potential bias because of the differences in the basic characteristics between the included and excluded patients and because of missing data.

Second, our database lacked information on previous vaccination status, the number of vaccine doses received, the viral load of COVID-19, and the resuscitation procedures implemented. We believe that incorporating these data would improve the robustness of validation of clinical status.

Third, this study predominantly included younger patients; 7,643 (82.3%) of our patients were aged younger than 65 years, and only 70 (0.75%) of these patients experienced mortality. Consequently, our results may not be directly applicable to other age groups. Therefore, we recommend that future studies conduct a separate analysis for patients aged 65 years and older. According to the literatures, comorbidities are one of the major risk factors for COVID-19 mortality. In our study, the prevalence of comorbidities was not high (< 3%). Although our results accurately reflect the situation in a real-world setting, the effects of COVID-19 on patients with multiple comorbidities should not be overlooked. Finally, vital signs were collected only once, upon each patient’s arrival at the ED. Obtaining data on prehospital vital signs and tracking a series of follow-up vital signs may increase the accuracy of predictions.

## Conclusion

Overall, the rSI-sMS is a simple and easily calculable tool that offers higher predictive performance for general ward admission, ICU admission, and mortality than do the SI and mSI among patients with COVID-19. Unlike other techniques that require complex charts or additional information or equipment, the rSI-sMS is a practical and real-time assessment tool that is applicable in both prehospital and ED settings; a lower rSI-sMS score indicates a higher risk of in-hospital mortality. These advantages render the rSI-sMS particularly valuable in resource-limited settings, such as in low- and middle-income countries.

### Supplementary Information

**Additional file 1: Supplementary Table 1. **Comparison of included sample and sample excluded because of missing vital sign data.

## Data Availability

The data used or analyzed in this study are available from the corresponding author upon reasonable request.
