# Supplementary material for: Reverse shock index multiplied by simplified motor score as a predictor of clinical outcomes for patients with COVID-19
Source: BMC Emerg Med. 2024 Feb 14;24:26. doi: 10.1186/s12873-024-00948-5 (PMC10865660; doi:10.1186/s12873-024-00948-5)
Supplement: Supplementary file 1 — Additional file 1: Supplementary Table 1. Comparison of included sample and sample excluded because of missing vital sign data. [file 12873_2024_948_MOESM1_ESM.docx]

**Supplementary Table 1.** Comparison of included sample and sample excluded because of missing vital sign data.

| **Characteristics** | Total | Numbers of missing record (%) | Included sample | Excluded sample | P-value |
| --- | --- | --- | --- | --- | --- |
| Patient number | 9282(100%) | 3.45% | 8961(96.55%) | 321(3.45%) |  |
| Age (years) |  |  |  |  |  |
| Age [median(IQR)] | 45(33-60) | 0% | 45(33-60) | 44(34-60) | 0.818 |
| Age <65ys | 7643(82.3%) | 0% | 7383(82.4%) | 260(81.0%) | 0.503 |
| Age ≥65ys | 1639(17.7%) | 0% | 1578(17.6%) | 61(19.0) |  |
| Sex, n (%) |  |  |  |  | 0.568 |
| Female | 5002(53.9%) | 0% | 4824(53.8%) | 178(55.5%) |  |
| Male | 4280(46.1%) | 0% | 4137(46.2%) | 143(44.5%) |  |
| Sepsis |  |  |  |  |  |
| SIRS score ≥ 2 | 5812(62.6%) | 0% | 5646(63.0%) | 166(51.7%) | <0.001 |
| SOFA score ≥ 2 | 160(1.7%) | 0% | 158(1.8%) | 2(0.6%) | 0.184 |
| Lactate ≥ 4.0 | 22(0.2%) | 0% | 23(0.3%) | 6(1.9%) | <0.001 |
| Comorbidity |  |  |  |  |  |
| CNS diseases | 230(2.5%) | 0% | 220(2.5%) | 10(3.1%) | 0.455 |
| CVD | 55(0.6%) | 0% | 55(0.6%) | 0(0.0%) | 0.159 |
| CKD | 68(0.7%) | 0% | 64(0.7%) | 4(1.2%) | 0.272 |
| Diabetes mellitus | 117(1.3%) | 0% | 116(1.3%) | 1(0.3%) | 0.121 |
| Outcomes |  |  |  |  |  |
| Admission | 660(7.11%) | 0% | 631(7.0%) | 29(9.0%) | 0.172 |
| ICU admission | 85(0.92%) | 0% | 76(0.8%) | 9(2.8%) | <0.001 |
| ED death | 9(0.1%) | 0% | 6(0.1%) | 3(0.9%) | <0.001 |
| In-hospital death | 70(0.75%) | 0% | 60(0.7%) | 10(3.1%) | <0.001 |
| Total LOS days | 9(5-15) | 0% | 9(5-15) | 10(6.5-14.0) | 0.889 |

CNS: central nervous system; CKD: chronic kidney disease; CVD: cardiovascular disease; SIRS: systemic inflammatory response syndrome; SOFA: Sequential Organ Failure Assessment; ED: emergency department; LOS: length of stay; ICU: intensive care unit.
